# Development and validation of a clinical-radiomics nomogram for differentiating *Mycoplasma pneumoniae* pneumonia from bacterial pneumonia in children

**DOI:** 10.3389/fped.2026.1764639

**Published:** 2026-07-08

**Authors:** Yan Guan, Xueqin Wang, Chen Song, Lulin Bi, Guang Yang, Shuai Quan, Shuming Xu

**Affiliations:** 1 Department of Medical Imaging, Children's Hospital of Shanxi, Taiyuan, China; 2 School of Medical Imaging, Shanxi Medical University, Taiyuan, China; 3 Department of Pediatrics, Shanxi Medical University, Taiyuan, China; 4 Medical Affairs, GE Healthcare (Shanghai) Co. Ltd., Shanghai, China

**Keywords:** bacterial pneumonia, children, machine learning, *Mycoplasma pneumoniae* pneumonia, nomogram, radiomics

## Abstract

**Objective:**

In this case-control study, we developed a nomogram merging computed tomography (CT)-based radiomics, clinical indicators, and CT imaging findings for differentiating *Mycoplasma pneumoniae* Pneumonia (MPP) from bacterial pneumonia (BP) in children.

**Methods:**

We retrospectively analyzed clinical and CT imaging data from 585 pediatric pneumonia patients, including 249 with MPP and 336 with BP. Patients were randomly allocated to training (70%) and validation (30%) groups. CT images were segmented using PHIgo-LK segmentation software (GE Healthcare), and radiomics features were extracted. The minimum redundancy maximum relevance (mRMR) and least absolute shrinkage and selection operator (LASSO) were used to screen the key features in the training group and the corresponding radiomics score were obtained. We developed three models: clinical, radiomics, and a combined nomogram model. Model performance was evaluated using the receiver operating characteristic (ROC) curve, calibration curves, and decision curve analysis.

**Results:**

The clinical model (variables: white blood cell count, C-reactive protein, lactate dehydrogenase, tree-fog sign, and bilateral lesions) achieved areas under the receiver operating characteristic curve (AUC) of 0.913 and 0.909 in the training and validation sets, respectively. The radiomics model built from five selected features reached AUCs of 0.918 and 0.895. Integration of clinical variables, CT morphology, and radiomics score into a nomogram delivered the highest accuracy, with AUCs of 0.971 and 0.958. Calibration curves confirmed the model's accuracy, and decision curve analysis highlighted significant net clinical benefit.

**Conclusion:**

The combined nomogram model could provide a decision-making basis for early clinical differentiation of MPP from BP in children.

## 1 Introduction

Community-acquired pneumonia (CAP) is a leading cause of morbidity and mortality in children worldwide (1, 2). In China, *Mycoplasma pneumoniae* is a predominant pathogen driving pediatric CAP, with particularly high incidence among school-age populations. Depending on epidemiological seasonality, *M. pneumoniae* pneumonia (MPP) accounts for 10%–40% of all CAP cases, and severe infections are often associated with extrapulmonary manifestations (3–5). Conversely, typical bacterial pneumonia (BP) is more common in young children, with *Streptococcus pneumoniae* remaining the most prevalent pathogen (6). However, in children aged 5 years and older, the incidence of *M. pneumoniae* infection is comparable to, or surpasses, that of typical bacterial pathogens (7, 8). Although timely pathogen identification is critical to guiding targeted antimicrobial therapy, the clinical presentations and imaging features of MPP and BP frequently overlap (9), making definitive differential diagnosis challenging.

The gold standard for pathogen identification in pneumonia is microbial culture; however, this approach is labor-intensive, time-consuming, highly susceptible to preanalytical variables, and frequently yields low positivity rates, thereby limiting its utility for early clinical diagnosis (10). Conversely, nucleic acid amplification testing for respiratory pathogens offers high sensitivity; however, remains relatively complex to perform and requires a specialized technical setup (11, 12). Similarly, mycoplasma-specific antibody testing is widely utilized in clinical practice, but diagnostic titers typically cannot be detected until one week following symptom onset (13). Consequently, chest computed tomography (CT) continues to play a vital role in acute evaluation; however, manual image interpretation is labor-intensive, inherently subjective, and heavily dependent on the clinician's experience and expertise. Furthermore, because the macrostructural imaging manifestations of different pathogenic pneumonias frequently exhibit a high degree of similarity (14), conventional visual inspection fails to swiftly, precisely, and efficiently differentiate between MPP and BP.

Recent studies have demonstrated that radiomics can extract high-dimensional, quantitative features from medical images that cannot be discerned by the naked eye (15). Consequently, radiomics-based approaches have been increasingly applied to inflammatory and infectious pulmonary diseases (16), demonstrating substantial utility in the classification, differential diagnosis, and prognostic assessment of coronavirus disease 2019 (COVID-19) (17–19). In addition, Deng et al. (20) proposed a deep learning model using a convolutional neural network to diagnose viral pneumonia from lung CT images, whereas Refaee et al. (21) highlighted the robust potential of high-resolution CT-based radiomics in identifying interstitial lung disease. These foundational advancements establish a compelling precedent and methodological framework for our current investigation.

In this study, we employed machine learning techniques to construct and validate a comprehensive diagnostic nomogram integrating CT radiomics signatures, clinical indicators, and visual CT features. This model aims to precisely differentiate pediatric MPP from BP, thereby establishing a reliable clinical decision-support tool to facilitate prompt and targeted therapeutic intervention.

## 2 Materials and methods

### 2.1 Patient selection

We retrospectively analyzed clinical and imaging data from pediatric patients diagnosed with single-pathogen MPP or BP at Shanxi Provincial Children's Hospital from November 2019 to February 2022.

Inclusion criteria included: (1) Confirmed MPP or BP diagnosis—where MPP defined by positive IgM serology (titer > 1:160) and/or Mycoplasma-specific PCR assay from respiratory specimens; patients with discordant laboratory findings (IgM-positive but PCR-negative) were classified as having probable *M. pneumoniae* infection and retained in the MPP cohort. BP was defined by a positive bacterial culture from sputum and/or blood. All laboratory tests were performed at admission, prior to the initiation of empirical antibiotic therapy. (2) Complete clinical records; (3) Pre-treatment chest CT scans with positive findings.

Exclusion criteria were: (1) Significant CT image artifacts; (2) Coinfection with two or more pathogens; (3) History of chronic pulmonary disease; (4) Concurrent immunodeficiency, hepatorenal disease, or connective tissue disorders.

**Figure 1:**
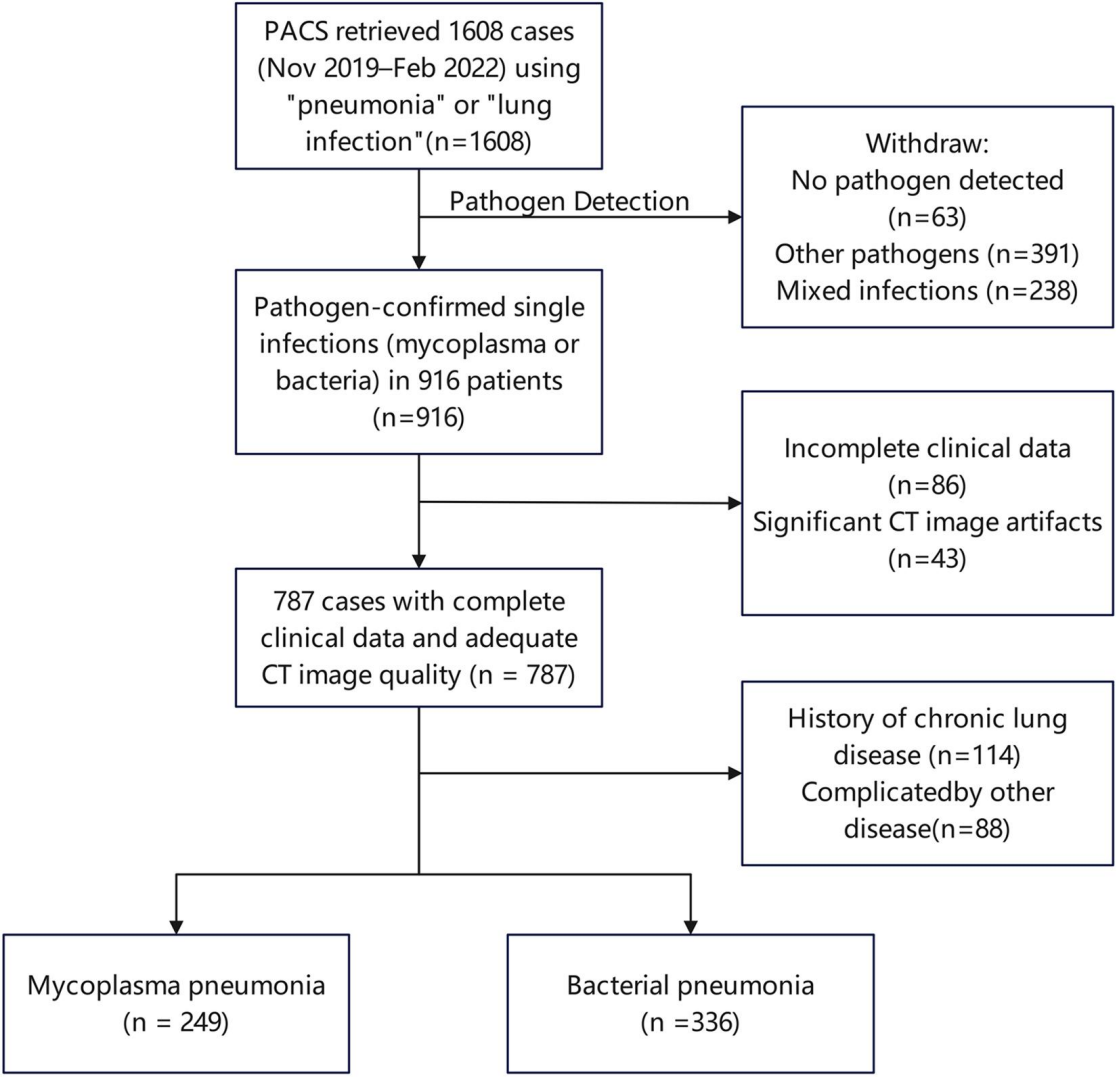
The process of selecting patients.

A total of 249 patients with MPP were enrolled, including 141 males and 108 females. Additionally, 336 patients with BP were enrolled, consisting of 203 males and 133 females (Figure 1).

### 2.2 Clinical data collection

For all included cases, the following medical records were collected from the hospital's electronic medical record system: (1) Demographics and clinical course: sex, age (years), time from symptom onset to admission (days), fever duration (days), and hospitalization time (days); (2) Clinical manifestations: maximum temperature (°C); presence of chest pain, dyspnea, rash, hypoxemia, and severe pneumonia; adventitious lung sounds (classified as absent, rales, rhonchi, or wheezing); and cough characteristics (classified as productive cough or nonproductive); (3) Laboratory parameters: white blood cell count (WBC, ×10^9^/L), neutrophil percentage (NEU%), lymphocyte percentage (LYM%), platelet count (PLT, ×10^9^/L), C-reactive protein (CRP, mg/L), procalcitonin (PCT, ng/mL), and lactate dehydrogenase (LDH, U/L).

### 2.3 Imaging data collection

All CT scans were performed using two scanners: SOMATOM Force CT (Siemens Healthcare, Forchheim, Germany) and Revolution CT (GE Healthcare, Waukesha, WI, USA). Scanning parameters were as follows: tube voltage, 100–120 kVp; tube current, adjusted by automatic exposure control; slice thickness, 1.25 mm; reconstruction interval, 0.5 mm; and pitch, 0.6–1.0. The raw CT data were subsequently transferred to the workstation for advanced analysis.

The initial CT scans were independently assessed by two senior radiologists, each with over 10 years of experience in diagnostic imaging. Interobserver agreement was assessed using Cohen's kappa statistic. In cases where discrepancies arose, a consensus was reached through discussion. The recorded CT imaging findings included the location and distribution of lesions, focal or multifocal lung consolidation, the tree-in-bud sign, the tree-fog sign, bronchial wall thickening, interlobular septal thickening, necrotic cavities, mediastinal or hilar lymphadenopathy, pleural effusion, and pericardial effusion.

### 2.4 Radiomics analysis

**Figure 2:**
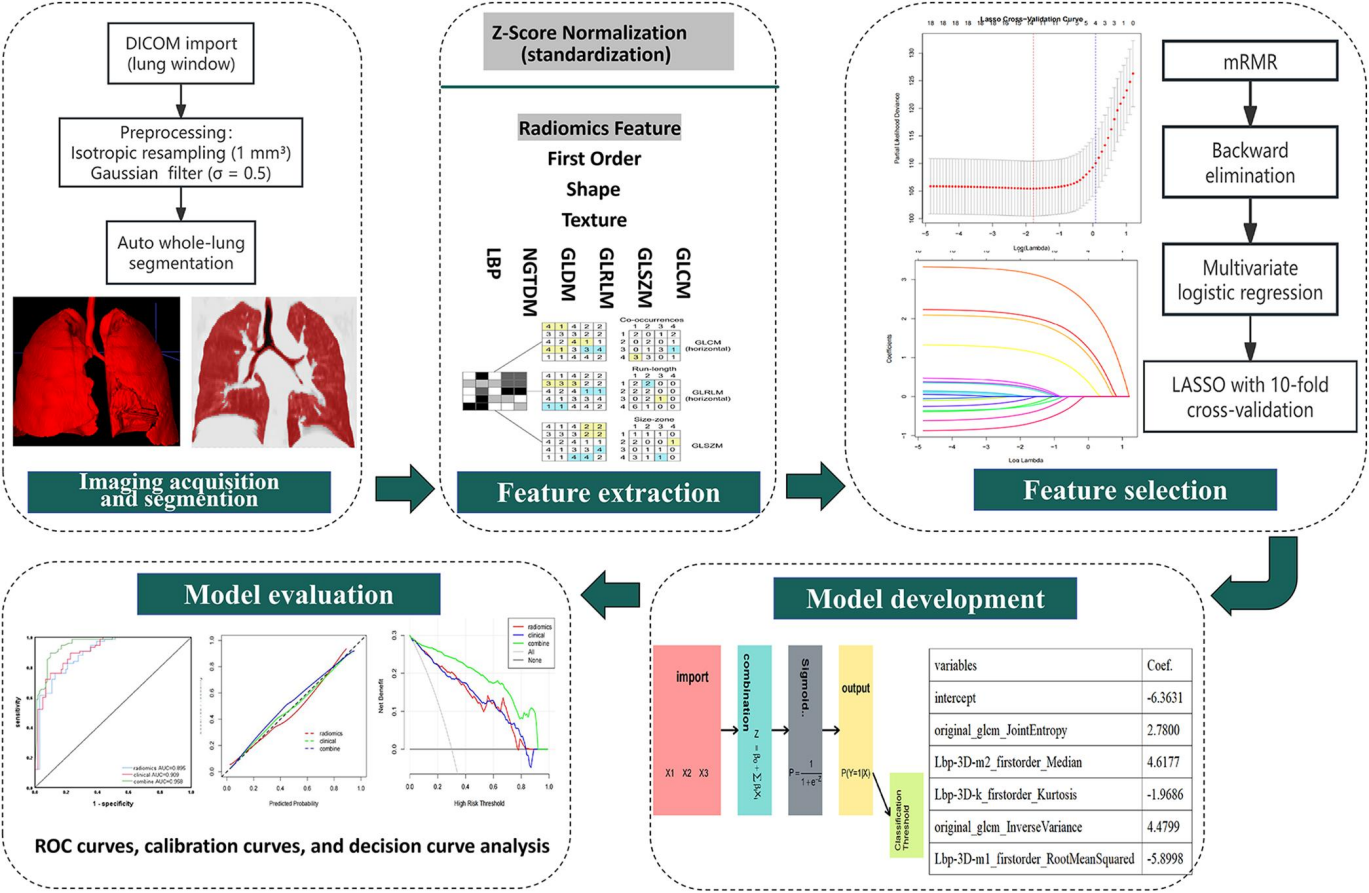
Radiomics workflow. (1) CT image preprocessing and automated whole-lung segmentation. (2) Following Z-score normalization, radiomics features were extracted: shape, first-order, and texture features (GLCM, GLSZM, GLRLM, GLDM, NGTDM and LBP). (3) Four-step feature selection: mRMR, backward elimination, multivariate logistic regression, and LASSO with 10-fold cross-validation. (4) Rad-score construction. (5) Model performance evaluation using ROC curves, calibration curves, and decision curve analysis.

As a central technical component of this study, radiomics analysis was used to extract quantitative medical imaging features and subsequently develop predictive models. Figure 2 illustrates the overall technical roadmap of the radiomics analysis, covering all steps from imaging data preprocessing to model construction and validation.

#### 2.4.1 Radiomics feature extraction

The original lung-window images from the initial CT scans of all cases were imported in Digital Imaging and Communications in Medicine format into the PHIgo-LK software (GE Healthcare, version 1.5.2) for segmentation. Preprocessing included resampling all CT images to an isotropic voxel size of 1 mm × 1 mm × 1 mm and applying a Gaussian filter (*σ* = 0.5) for denoising; subsequently, automatic whole-lung segmentation was performed to define the volume of interest (VOI). The VOI encompassed the entire lung parenchyma, including intrapulmonary vasculature and bronchi, rather than individual segmentation of focal pneumonic lesions. This approach captures global textural and density patterns across the lung tissue (22–24), which often exhibit a diffuse or multifocal distribution during infectious pulmonary processes.

The segmentation results were manually reviewed and corrected by a radiologist with 10 years of experience in chest imaging diagnostics, and subsequently verified by another radiologist with 15 years of experience in the same field. Before feature extraction, z-score standardization was applied to standardize voxel intensities across the original images, transforming the gray-level values to conform to a standard normal distribution.

Radiomics features were extracted from the segmented VOI using PyRadiomics (version 3.1.0), an open-source Phyton package. A total of 386 radiomics features were extracted from each patient, encompassing the following categories: 14 shape features, 18 first-order statistics, 24 gray-level co-occurrence matrix (GLCM) features, 16 gray-level size-zone matrix features, 16 gray-level run-length matrix (GLRLM) features, 14 gray-level dependence matrix features, 5 neighboring gray tone difference matrix features, and 279 features derived from the three-dimensional local binary pattern (3D-LBP) filter.

#### 2.4.2 Radiomics feature selection and modeling

The 585 patients were randomly divided into a training set (*n* = 410) and a validation set (*n* = 175) in a 7:3 ratio using a simple random sampling method. The training set was used for feature screening and model construction, while the validation set was used exclusively for model validation.

To manage the high dimensionality of the radiomics features and ensure model stability, a sequential feature selection strategy was implemented. Initially, within the training group, the maximum relevance minimum redundancy (mRMR) algorithm was applied to remove redundant and irrelevant features from the radiomics dataset. This step served to reduce computational burden and eliminate noise prior to statistical modeling. Subsequently, backward elimination was employed to exclude non-significant predictors. The retained features were then subjected to multivariable logistic regression analysis to confirm their statistical significance in a multivariable context. Finally, to prevent overfitting and refine the feature set, the least absolute shrinkage and selection operator (LASSO) regression model with 10-fold cross-validation was used to determine the optimal hyperparameter (λ) and ultimately select features with nonzero coefficients to construct the radiomics signature.

### 2.5 Clinical and combined nomogram model construction

#### 2.5.1 Clinical model

Variables with *P* < 0.05 in univariable logistic regression were entered into multivariable logistic regression. Backward elimination was performed to identify independent predictors, which were then incorporated into the clinical model.

#### 2.5.2 Combined nomogram model

The radiomics score (Rad-score) obtained from Section 2.4.2 was integrated with the independent predictors in the clinical model using multivariable logistic regression. The final combined nomogram was constructed based on the regression coefficients of the selected variables.

#### 2.5.3 Model evaluation

The performance of all three models (clinical, radiomics, and combined nomogram) was evaluated using the following metrics: area under the receiver operating characteristic curve (AUC), sensitivity, specificity, positive predictive value, negative predictive value (NPV), and accuracy with 95% confidence intervals. Calibration curves were generated to assess the agreement between predicted and observed probabilities. Decision curve analysis (DCA) was performed to evaluate the net clinical benefit of the models at different threshold probabilities. The DeLong test was used to compare the AUCs of different models.

### 2.6 Statistical analysis

Statistical analyses were conducted using SPSS version 26.0 and R version 3.6.0. The normality of continuous variables was evaluated using the Kolmogorov–Smirnov test. Normally distributed data were expressed as mean ± standard deviation, and comparisons between groups were performed using the independent samples *t*-test. Non-normally distributed data were expressed as median with interquartile range (IQR), and the Mann–Whitney *U* test was applied for group comparisons. Categorical variables were expressed as frequencies and percentages, and analyzed using the chi-squared test, where appropriate. The LASSO algorithm was performed using the “glmnet” package in R software. Logistic regression modeling and calibration curve analysis were conducted with the “rms” package. Receiver operating characteristic (ROC) curve and DCA were performed using the “rmda” package. Multivariable regression and feature selection were carried out using the “mRMRe” package. *P* < 0.05 was considered significant.

## 3 Results

### 3.1 Baseline characteristics

A total of 585 pediatric patients diagnosed with MPP or BP were enrolled and split into a training cohort (*n* = 410; 170 MPP vs. 240 BP) and a validation cohort (*n* = 175; 79 MPP vs. 96 BP). In the training set, WBC counts and interlobular septal thickening differed significantly between groups. In the validation set, age, neutrophil percentage (NEU%) and lymphocyte percentage (LYM%), adventitious lung sounds, and time from symptom onset to admission were significantly different. Across both cohorts, fever duration and maximum temperature differed significantly between the two groups (all *P* < 0.05; exact values provided in Supplementary Table S1).

### 3.2 Interobserver agreement

Interobserver agreement for all 13 CT imaging findings was substantial to almost perfect, with Cohen's kappa values ranging from 0.712 to 0.978 (Supplementary Table S2).

### 3.3 Clinical model development and performance

**Figure 3:**
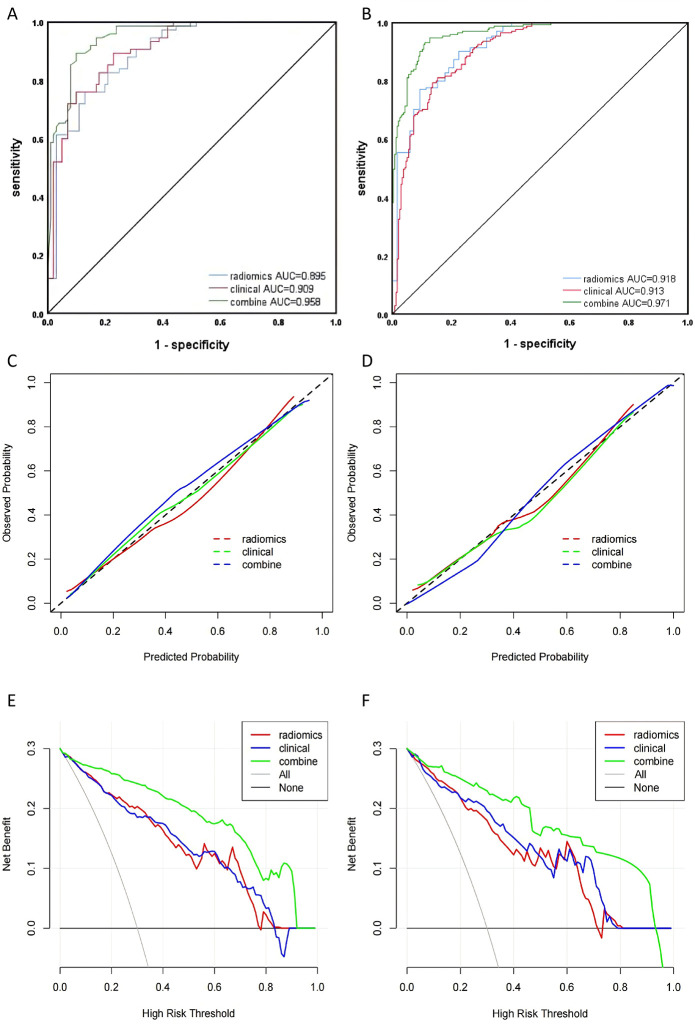
Diagnostic performance of the three models. **(A)** ROC curve of the training set; **(B)** ROC curve of the validation set; **(C)** Calibration curve of the training set; **(D)** Calibration curve of the validation set; **(E)** Decision curve of the training set; **(F)** Decision curve of the validation set.

Univariable analysis identified five variables that significantly distinguished MPP from BP: lactate dehydrogenase (LDH), WBC count, CRP, the tree-fog sign, and bilateral lesions (Table 1). All remained independently associated in multivariable logistic regression (*P* < 0.05) and were incorporated into the clinical model. The model achieved AUCs of 0.913 (95% CI, 0.696–0.925) in the training cohort and 0.909 (95% CI, 0.854–0.952) in the validation cohort (Figure 3).

**Table 1:** Univariate and multivariate regression results.

Characteristic	Univariate analysis		Multivariate analysis	
	OR (95%CI)	*P* value	OR (95%CI)	*P* value
Sex (male)	1.268 (0.645–2.493)	0.234		
Age	0.429 (0.213–0.865)	0.096		
Hypoxemia	0.570 (0.293–1.108)	0.321		
Severe Pneumonia	1.465 (0.695–3.087)	0.079		
Time to admission	1.374 (0.708–2.668)	0.095		
Hospitalization time	0.789 (0.401–1.551)	0.545		
Fever duration	0.994 (0.450–2.193)	0.164		
Maximum temperature	2.364 (0.500–11.200)	0.974		
Adventitious lung sounds	0.753 (0.292–1.947)	0.164		
Cough Type	1.807 (0.929–3.517)	0.342		
Chest pain	0.247 (0.107–0.567)	0.085		
Dyspnea	2.098 (0.894–4.920)	0.168		
Hemoptysis	2.406 (1.041–5.562)	0.125		
Rash	2.518 (1.182–5.364)	0.164		
WBC	0.440 (0.252–0.770)	0.004	0.872 (0.830–0.916)	<0.001
NEU%	0.649 (0.381–1.104)	0.063		
LYM%	3.765 (4.767–5.975)	0.532		
PLT	0.669 (0.049–9.227)	0.764		
CRP	0.953 (0.921–0.986)	0.005	0.994 (0.987–1.000)	0.042
PCT	1.746 (0.900–3.400)	0.534		
LDH	1.746 (1.324–2.213)	0.013	1.001 (1.000–1.003)	0.030
Pleural effusion	0.440 (0.183–1.061)	0.749		
Pericardial effusion	0.649 (0.381–1.104)	0.193		
Atelectasis	3.134 (0.935–6.955)	0.097		
Consolidation extent	0.827 (0.402–1.701)	0.294		
Pulmonary necrosis	1.167 (0.497–2.740)	0.723		
Bronchial wall thickening	0.993 (0.641–1.538)	0.975		
Interlobular septal thickening	1.305 (0.933–1.824)	0.120		
Tree-in-bud sign	0.772 (0.540–1.103)	0.155		
Tree-fog sign	0.211 (0.148–0.300)	<0.001	0.143 (0.093–0.220)	<0.001
Mediastinal or Hilar lymph node enlargement	1.070 (0.612–1.873)	0.812		
Left upper lobe	1.367 (0.982–1.904)	0.064		
Left lower lobe	1.100 (0.792–1.527)	0.570		
Right upper lobe	1.102 (0.793–1.530)	0.563		
Right middle lobe	1.141 (0.815–1.597)	0.444		
Right lower lobe	1.288 (0.923–1.795)	0.136		
Left lung	1.217 (0.857–1.728)	0.272		
Right lung	1.215 (0.807–1.828)	0.350		
Bilateral lungs	2.086 (1.494–2.913)	<0.001	3.836 (2.494–5.898)	<0.001

CRP, C-reactive protein; LYM%, lymphocyte ratio; LDH, lactic dehydrogenase; M (IQR), median interquartile range; M (SD), median standard deviation; NEU%, neutrophil ratio; PCT, procalcitonin; PLT, platelet; WBC, white blood cell.

### 3.4 Radiomics model development and performance

From the initial 386 extracted radiomics features, the mRMR algorithm was first applied to select 80 candidate features with the strongest relevance to the outcome variable and minimal mutual redundancy. Subsequently, backward elimination was performed to further reduced the feature pool to 62 features. Multivariable logistic regression analysis was then conducted on these 62 features, ultimately retaining 18 features. Finally, to eliminate collinearity and prevent overfitting, LASSO regression with 10-fold cross-validation was employed to apply an *L*_1_ regularization penalty. The optimal penalty parameter (λ) was determined at the minimum cross-validation error, compressing the coefficients of redundant features to zero and ultimately yielding 5 optimal features with nonzero coefficients to construct the final radiomics signature (Supplementary Figure S1). These features were linearly combined based on their respective coefficient weights to calculate the radiomics score (Rad-score) for each subject as follows:

$$\begin{array}{rl} \mathrm{Rad}\text{-}\mathrm{score}\;= & -6.3631\;+\;2.7800\;\times \;\mathrm{original}\_\mathrm{glcm}\_\mathrm{JointEntropy}\\ & +\;4.6177\;\times \;\mathrm{Lbp}\text{-}3D\text{-}m2\_\mathrm{firstorder}\_\mathrm{Median}\\ & -\;1.9686\times \;\mathrm{Lbp}\text{-}3D\text{-}k\_\mathrm{firstorder}\_\mathrm{Kurtosis}+4.4799\\ & \times \mathrm{original}\_\mathrm{glcm}\_\mathrm{InverseVariance}\;-\;5.8998\\ & \times \;\mathrm{Lbp}\text{-}3D\text{-}m1\_\mathrm{firstorder}\_\mathrm{RootMeanSquared} \end{array}$$

The AUC values of the radiomics model in the training and validation cohorts were 0.918 and 0.895, respectively (Figure 3).

### 3.5 Combined nomogram model

**Figure 4:**
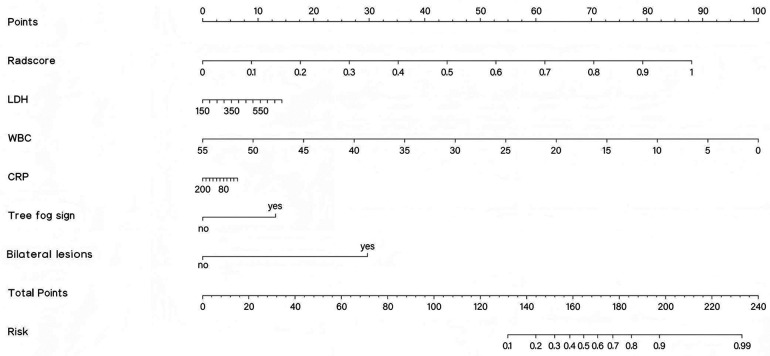
Nomogram model for differentiating MP from BP in children.

The combined nomogram model was developed by integrating the Rad-score with the clinical model (Figure 4). This integrated approach demonstrated superior diagnostic discrimination, achieving AUC values of 0.971 for the training cohort and 0.958 for the validation cohort (Figure 3). DeLong test comparisons confirmed that the combined nomogram performed significantly better than the clinical model and radiomics model (all *P* < 0.001), whereas no significant difference was observed between the clinical and radiomics models (*P* = 0.562 and *P* = 0.327 in the training and validation cohorts, respectively).

The calibration curve and DCA demonstrated that the combined nomogram model exhibited superior performance compared to the clinical model and the radiomics model (Figure 3), indicating a high clinical net benefit. The Hosmer–Lemeshow test indicated no significant lack of fit for the radiomics model (training: *P* = 0.556; validation: *P* = 0.491), clinical model (training: *P* = 0.247; validation: *P* = 0.329), or combined nomogram (training: *P* = 0.652; validation: *P* = 0.781).

The diagnostic efficiency of the three models was also evaluated on the training and validation sets. As detailed in Table 2, the combined nomogram exhibited the best overall diagnostic efficacy, as evidenced by its highest AUC, accuracy, and F1 score. While the standalone radiomics model achieved the highest sensitivity, and the clinical model the highest specificity and NPV, the nomogram provided the most balanced and robust performance profile across all evaluated metrics.

**Table 2:** Diagnostic efficiency of three models in train and validation set.

Information	Clinical model		Radiomics model		Combined model	
	Training set	Validation set	Training set	Validation set	Training set	Validation set
AUC	0.913 (0.696,0.925)	0.909 (0.854, 0.952)	0.918 (0.885, 0.946)	0.895 (0.842, 0.938)	0.971 (0.942, 0.972)	0.958 (0.938, 0.986)
Accuracy	0.883 (0.848, 0.913)	0.854 (0.794, 0.902)	0.854 (0.817, 0.887)	0.834 (0.771, 0.884)	0.888 (0.860, 0.915)	0.867 (0.810, 0.910)
F1_Score	0.303 (0.251, 0.359)	0.286 (0.215, 0.367)	0.881 (0.850, 0.905)	0.855 (0.800, 0.895)	0.890 (0.860, 0.915)	0.875 (0.820, 0.915)
Sensitivity	0.185 (0.149, 0.226)	0.178 (0.124, 0.246)	0.887 (0.853, 0.915)	0.856 (0.794, 0.904)	0.834 (0.795, 0.868)	0.831 (0.767, 0.883)
Specificity	0.994 (0.979, 0.998)	0.987 (0.956, 0.997)	0.854 (0.817, 0.887)	0.832 (0.769, 0.882)	0.953 (0.930, 0.970)	0.912 (0.859, 0.949)
Positive prediction	0.833 (0.747, 0.897)	0.714 (0.579, 0.822)	0.875 (0.840, 0.906)	0.854 (0.792, 0.902)	0.955 (0.934, 0.971)	0.923 (0.872, 0.957)
Negative prediction	0.885 (0.851, 0.913)	0.860 (0.800, 0.905)	0.854 (0.817, 0.887)	0.832 (0.769, 0.882)	0.845 (0.807, 0.878)	0.963 (0.922, 0.984)

## 4 Discussion

Despite marked improvements in the treatment outcomes and cure rates, CAP remains the leading cause of infectious disease mortality among Chinese children (25). Timely pathogen diagnosis is crucial to prevent antibiotic overuse and the development of drug resistance. Given that delayed diagnosis elevates also the risk of irreversible respiratory damage (26, 27), early and precise etiological identification is essential.

Clinicians typically rely on clinical symptoms, imaging features, and inflammatory markers for empirical pathogen identification to formulate treatment plans. In this retrospective study, we developed a machine learning model integrating CT-based radiomic signatures, clinical factors, and CT imaging findings to differentiate the most common forms of CAP in children, namely MPP and BP.

### 4.1 Clinical indicators and CT imaging findings for differentiating MPP from BP

In clinical practice, traditional inflammatory markers, including WBC count, neutrophil percentage, and CRP levels, remain widely utilized for diagnosing infections such as pneumonia, despite their limitations (28, 29). Although more specific and sensitive markers like CD64 have been developed (30), their application in clinical settings is limited due to the requirement for specialized equipment and technical expertise, thus they are primarily employed in research contexts. In our study, WBC counts and CRP levels were found to be mildly elevated in patients with MPP, yet significantly lower than those observed in patients with BP, indicating a statistically significant difference. This difference may be attributed to the more robust inflammatory response associated with BP, alongside the strict exclusion criteria applied to our study cohort, which minimized potential confounding factors. Additionally, our findings revealed higher LDH levels—a crucial indicator of cellular injury—in children with MPP compared to those with BP. This aligns with the well-established finding that *M. pneumoniae* infection frequently damages extrapulmonary tissues (5, 31, 32).

In the early stages of MPP in children, the radiological findings are indistinguishable from those of lobular BP (bronchopneumonia). As the disease progresses to more extensive consolidation, the CT features closely mimic those of lobar BP. Although we found no statistically significant differences in the CT features of bronchial wall thickening, tree-in-bud signs, centrilobular nodules, lobular distribution, or segmental solid lesions between MPP and BP, the tree-fog sign was more frequently observed in patients with MPP rather than BP, a finding that aligns with prior research (33). This is likely attributable to inflammatory and exudative alterations within the peribronchial interstitium (Supplementary Figure S2). Therefore, the presence of the tree-fog sign may help to differentiate between MPP and BP.

Radiographically, MPP presented as bilateral, multifocal, and irregular patchy ground-glass opacities interspersed with areas of consolidation, predominantly affecting the lower lobes. This pattern is consistent with the findings reported by Huang et al. (9). By contrast, BP typically affects a single lung and is characterized by large areas of confluent alveolar infiltration or consolidated lesions (Supplementary Figure S2). Therefore, identifying a bilateral, multi-lobar lesion distribution alongside the tree-fog sign may facilitate the differential diagnosis between MPP and BP.

The clinical model incorporated five diagnostic indicators: WBC, CRP, LDH, tree fog sign, and bilateral lung disease, yielding AUCs of 0.913 in the training set and 0.909 in the validation set. However, despite these high AUC values, the model exhibited high specificity (0.994) but limited sensitivity (0.185). This imbalance stems from the non-specific nature of the selected variables, which fail to capture MPP-specific pathophysiology. Consequently, the clinical model has only modest value for distinguishing MPP from BP, underscoring the necessity of radiomics integration for reliable differential diagnosis.

### 4.2 Role of CT-based radiomics in the differential diagnosis of MPP and BP

MPP and BP exhibit similar features on CT, making differentiation challenging based solely on radiologists' visual inspection and clinical experience. However, radiomics can enhance diagnostic accuracy by quantifying lesion characteristics and revealing subtle internal differences (15–19, 34). Unlike most previous radiomics studies employing lesion-based segmentation, we adopted a whole-lung segmentation approach. This decision was primarily driven by the patchy, multifocal, and coalescent nature of MPP and BP, in which precise lesion delineation is often challenging and subject to high interobserver variability. Whole-lung segmentation circumvents these issues and enables characterization of global disease distribution patterns, such as spatial heterogeneity and lobar predominance, that may contain diagnostic information beyond focal lesion features (35). However, this approach also has inherent limitations. By incorporating large volumes of normal lung parenchyma, whole-lung segmentation may dilute disease-specific radiomic signals and introduce nonspecific feature contributions from nonpathological tissue, potentially reducing the biological specificity and interpretability of the extracted features (36). For the heterogeneous pneumonia cases in this study, whole-lung segmentation offered improved robustness and reproducibility, albeit at the expense of some pathological specificity. Future comparative studies are warranted to determine the optimal segmentation strategy for this disease entity.

The machine learning technique employed in our study to analyze CT-based radiomics features successfully identified five key features that distinguish between MPP and BP. These features comprised two GLCM features, indicative of spatial density and tissue heterogeneity alterations, and three LBP features, reflecting detailed microstructural differences in the lungs. The resulting radiomics model showed high discriminatory power on the training and validation sets.

### 4.3 Clinical value of the combined nomogram model

To enhance the diagnostic model's effectiveness, this study integrated the Rad-score with the clinical model to construct a nomogram. The combined model demonstrated superior diagnostic capability, with AUC values of 0.971 and 0.958 for the training and validation groups, respectively. To rigorously assess the superiority of the combined model, pairwise comparisons of the ROC curves were performed using the DeLong test. The results confirmed that the clinical-radiomics nomogram yielded significantly higher AUC values than either the clinical model or the radiomics model in the training and validation sets (*P* < 0.001 for all comparisons). In contrast, the difference in AUC between the clinical model and the radiomics model alone was not statistically significant in either cohort. These findings robustly support our of hypothesis that integrating radiomics features with clinical parameters provides complementary diagnostic information. The combination of clinical features, which capture the patient's macroscopic physiological state, with radiomics features, which uncover the microscopic heterogeneity of the lesion, yields a more comprehensive and robust predictive model. The comparable performance of the clinical and radiomics models suggests that, for the current diagnostic task, neither single information source is sufficient to fully characterize the complexity of the disease. This further highlights the necessity of multimodal information fusion.

The calibration curve and DCA confirmed the nomogram's good reliability and stability, along with higher clinical net benefits. In addition to predictive accuracy, good calibration is crucial for clinical applicability; the Hosmer–Lemeshow test results confirmed that all three models exhibited satisfactory calibration in the training and validation sets (all *P* > 0.05). This indicates that the probabilities generated by our models reliably reflected the true likelihood of the event, which is a key strength supporting their potential utility in clinical decision-making. Moreover, the nomogram's visual representation facilitates easier comprehension and acceptance by clinicians and patients, which is objective and compelling.

To our knowledge, the diagnostic efficiency of the combined model surpasses that of previous similar studies (37–39). Wang et al. (40) developed a radiomics nomogram to differentiate MPP from *S. pneumoniae* pneumonia (AUC 0.822) using focal ROI delineation (consolidation ± halo). Chen et al. (41) recognized necrotizing pneumonia using lesion-based radiomics (AUC 0.76). In contrast, our study employed whole-lung segmentation rather than focal lesion delineation. This strategy is designed to capture the diffuse and multifocal parenchymal involvement often seen in pediatric pneumonia, potentially providing a more comprehensive representation of the disease' imaging phenotype. Whereas both previous studies relied on radiomics-only nomograms, we developed a multimodal nomogram integrating radiomics signatures, clinical indicators, and CT imaging findings, demonstrating that diverse data sources synergistically enhance diagnostic accuracy.

### 4.4 Limitations

Several limitations of our study should be considered. First, the retrospective design and single-center data collection may introduce some component of selection bias. Although internal validation was performed, both cohorts were derived from the same institution, limiting generalizability. Second, although standardized preprocessing was applied within this center, the model's generalizability to external scanners, protocols, and patient populations remains unvalidated. Third, although the combined model demonstrated robust performance across the training and validation cohorts, internal resampling procedures—such as bootstrapping or repeated cross-validation—were not performed to further evaluate model stability and protect against overfitting. Fourth, radiomics feature reproducibility was not formally assessed. Fifth, in this study, we did not differentiate among bacterial species or include viral and mixed infections. Future prospective multicenter studies should incorporate additional resampling methods, reproducibility analyses, and broader infectious etiologies to validate these findings.

## 5 Conclusion

In summary, the machine-learning diagnostic model utilizing chest CT radiomic signatures presents a novel approach for distinguishing MPP from BP in children. Furthermore, the multimodal nomogram incorporating the Rad-score, clinical indicators, and conventional CT features demonstrates improved diagnostic performance, offering enhanced clinical utility for optimizing clinical management.

## Data Availability

The datasets presented in this article are not readily available due to patient privacy and institutional ethics requirements, the raw data underlying this study. De-identified data may be shared with qualified researchers upon reasonable request and with appropriate ethical approvals. Requests to access the datasets should be directed to the corresponding author.

## Supplementary material

Supplementary Figure S1 Tuning parameter selection using the LASSO regression. **(A)** Each colored line represents a clinical feature’s coefficient as a function of log(λ). As λ increases, coefficients shrink towards zero, highlighting feature selection. Five features with nonzero coefficients at the optimal λ were selected. By verifying the optimal parameter (λ) in the LASSO model. The partial likelihood binomial deviance is plotted vs log (λ) and dotted vertical lines were set using the minimum criteria and the one standard error of the minimum criteria **(B)**.

Supplementary Figure S2 **(A)** Chest CT of bacterial pneumonia (*Streptococcus pneumoniae*). Male, 62 months old, CRP 72 mg/L, WBC 12.8 × 10^9^/L, LDH 380 U/L. Large areas of consolidation in the right upper lobe with visible air bronchograms. **(B)** Chest CT of mycoplasma pneumonia. Male, 56 months old, CRP 19 mg/L, WBC 10.9 × 10^9^/L, LDH 291 U/L. Small patchy consolidations in both lower lobes and peribronchial ground-glass opacities (tree fog sign) in the right lower lobe.
