# Supplementary material for: Development and validation of a clinical-radiomics nomogram for differentiating Mycoplasma pneumoniae pneumonia from bacterial pneumonia in children
Source: Front Pediatr. 2026 Jul 8;14:1764639. doi: 10.3389/fped.2026.1764639 (PMC13388284; doi:10.3389/fped.2026.1764639)
Supplement: Supplementary Figure S1 — Tuning parameter selection using the LASSO regression. (A) Each colored line represents a clinical feature’s coefficient as a function of log(λ). As λ increases, coefficients shrink towards zero, highlighting feature selection. Five features with nonzero coefficients at the optimal λ were selected. By verifying the optimal parameter (λ) in the LASSO model. The partial likelihood binomial deviance is plotted vs log (λ) and dotted vertical lines were set using the minimum criteria and the one standard error of the minimum criteria (B). [file Table1.docx]

Table S1 Comparison of baseline characteristics of patients in the training and validation cohorts

| Characteristic | Training cohort | | | |  | Validation cohort | | | |
| --- | --- | --- | --- | --- | --- | --- | --- | --- | --- |
|  | MP（n=170） | BP（n=240） | Statistical Value | *P* value |  | MP（n=79） | BP（n=96） | Statistical Value | *P* value |
| Sex (male) , n(%) | 95(55.9%) | 143 (59.6%） | 0.558 | 0.455 |  | 46 (58.2%) | 60(62.5%） | 0.329 | 0.566 |
| Age (months), M (IQR) | 60.00 (69.25) | 72.00 (74.75) | 1.427 | 0.232 |  | 31.00(52.00) | 84.00(60.00) | 37.626 | <0.001 |
| Time to admission, M (IQR) | 5.00 (4.00, 9.00) | 5.00 (3.00, 9.25) | 2.840 | 0.092 |  | 7.00 (4.00, 12.00) | 5.00 (3.00, 10.00) | 6.850 | 0.009 |
| **Symptoms and signs, n (%)** |  |  |  |  |  |  |  |  |  |
| Maximum temperature | 38.90 (37.50, 39.63) | 38.65 (37.00, 39.50) | 6.302 | 0.012 |  | 38.30 (39.20, 39.80) | 38.50 (36.90, 39.00) | 12.672 | <0.001 |
| Chest pain | 1（0.6%） | 1（0.4%） | 0.060 | 0.806 |  | 1 (1.3%) | 1（1.0%） | 0.019 | 0.890 |
| dyspnea | 14（8.2%） | 18（7.5%） | 0.075 | 0.785 |  | 10 (12.7%) | 8 (8.3%) | 0.873 | 0.350 |
| Rash | 10 (5.9%) | 14（5.8%） | 0.000 | 0.983 |  | 6 (7.6%) | 4 (4.2%) | 0.940 | 0.332 |
| Hypoxemia | 21 (12.4%) | 40（16.7%） | 1.459 | 0.227 |  | 7 (8.9%) | 17 (17.7%) | 2.850 | 0.091 |
| Severe Pneumonia | 46 (27.1%) | 73（30.4%） | 0.543 | 0.461 |  | 17 (21.5%) | 23 (24.0%) | 0.145 | 0.703 |
| Adventitious lung sounds |  |  | 2.350 | 0.125 |  |  |  | 7.224 | 0.007 |
| Absent | 49 (28.8%) | 59（24.6%） |  |  |  | 34 (43.0%) | 19 (19.8%) |  |  |
| Rales | 83 (48.8%) | 110（45.8%） |  |  |  | 27 (34.2%) | 46 (47.9%) |  |  |
| Rhonchi | 28 (16.5%) | 54（22.5%） |  |  |  | 13 (16.5%) | 27 (28.1%) |  |  |
| Wheezing | 10 (5.9%) | 17（7.1%） |  |  |  | 5 (6.3%) | 4 (4.2%) |  |  |
| Cough Type |  |  | 0.286 | 0.593 |  |  |  | 1.390 | 0.238 |
| Chesty cough | 141 (82.9%) | 192（80.0%） |  |  |  | 60 (75.9%) | 85 (88.5%) |  |  |
| Dry cough | 26 (15.3%) | 41（17.1%） |  |  |  | 14 (17.7%) | 9 (9.4%) |  |  |
| **Outcomes** |  |  |  |  |  |  |  |  |  |
| Fever duration, M (IQR),d | 5.00 (2.00, 8.00) | 3.00 (1.00, 6.00) | 13.877 | <0.001 |  | 5.00 (2.00, 7.00) | 3.00 (1.00, 6.00) | 5.302 | 0.021 |
| Hospitalization time, M (IQR), d | 8.00 (7.00, 11.00) | 7.00 (7.00,10.00) | 1.517 | 0.218 |  | 7.00 (7.00,10.00) | 8.00 (7.00, 10.00) | 0.127 | 0.721 |
| **Laboratory values** |  |  |  |  |  |  |  |  |  |
| WBC, M (IQR), 10^9^/L | 9.98 (7.61,  13.61) | 11.46 (8.15,14.43) | 7.531 | 0.006 |  | 11.54 (6.54, 13.53) | 11.21 (8.02,14.11) | 2.024 | 0.155 |
| NEU% | 51.95 (32.73, 67.75) | 50.65 (32.13, 66.48) | 1.899 | 0.168 |  | 61.35 (39.48, 72.83) | 45.40 (29.20, 66.30) | 9.281 | 0.002 |
| LYM% | 39.34±19.05 | 36.43±17.97 | -1.559 | 0.116 |  | 28.15 (19.70, 45.15) | 41.90 (24.45, 59.80) | 13.491 | <0.001 |
| PLT, M(IQR), 10^9^/L | 328.50 (265.75, 421.00) | 343.50 (258.00, 435.00) | 2.273 | 0.132 |  | 320.00 (220.00, 426.00) | 351.00 (289.50, 437.50) | 2.744 | 0.098 |
| CRP, M (IQR), mg/L | 7.89 (1.51,  27.78) | 7.81 (1.93,  26.15) | 0.077 | 0.782 |  | 11.04 (2.73, 36.33) | 6.37 (1.21, 33.71) | 1.547 | 0.214 |
| PCT, M (IQR), ng/ml | 0.08 (0.04,  0.39) | 0.08 (0.05,  0.29) | 0.504 | 0.478 |  | 0.11 (0.05, 0.42) | 0.13 (0.05,  0.50) | 0.101 | 0.751 |
| LDH , M (IQR), U/L | 307.50 (266.75, 367.25) | 301.00 (253.75, 350.25) | 0.425 | 0.514 |  | 293.00 （242.75, 342.25） | 298.00 (264.50, 345.00） | 2.610 | 0.106 |
| **Radiological features , n(%)** |  |  |  |  |  |  |  |  |  |
| Lesion site |  |  |  |  |  |  |  |  |  |
| Left lung | 125 (73.5%) | 167（69.9%） | 0.648 | 0.421 |  | 53 (67.1%) | 66 (68.8%) | 0.113 | 0.737 |
| Right lung | 145 (85.3%) | 191（79.9%） | 1.955 | 0.162 |  | 59 (74.7%) | 81 (84.4%) | 3.053 | 0.081 |
| Bilateral lungs | 108 (63.5%) | 147（61.5%） | 0.173 | 0.678 |  | 41 (51.9%) | 61 (63.5%) | 2.680 | 0.102 |
| Bronchial wall thickening | 140 (82.4%) | 202（84.2%） | 0.236 | 0.627 |  | 62 (78.5%) | 77 (80.2%) | 0.079 | 0.779 |
| Interlobular septal thickening | 71 (41.8%) | 60（25.0%） | 12.832 | <0.001 |  | 32 (40.5%) | 37 (38.5%) | 0.070 | 0.792 |
| Tree-in-bud sign | 100 (58.8%) | 144（60.0%） | 0.057 | 0.811 |  | 48 (60.8%) | 65 (67.7%) | 0.909 | 0.304 |
| Tree-fog sign | 61 (35.9%) | 67（28.0%） | 2.839 | 0.092 |  | 31 (39.2%) | 26 (27.1%) | 2.744 | 0.098 |
| Mediastinal or Hilar lymph node enlargement | 21 (12.4%) | 25（10.4%） | 0.374 | 0.541 |  | 5 (6.3%) | 8 (8.3%) | 0.252 | 0.616 |
| Consolidation extent |  |  | 1.618 | 0.203 |  |  |  | 0.172 | 0.679 |
| No pulmonary consolidation | 78 (45.9%) | 94 (39.2%) |  |  |  | 34 (43.0%) | 38 (39.6%) |  |  |
| single lung segment | 44 (25.9%) | 69（28.7%) |  |  |  | 24 (30.4%) | 31 (32.3%) |  |  |
| multiple lung segment | 48 (28.2%) | 77（32.1%) |  |  |  | 21 (26.6%) | 27 (28.1%) |  |  |
| Pleural effusion | 22（12.9%） | 40（20.0%） | 3.492 | 0.062 |  | 13 (16.5%) | 8 (8.3%) | 2.692 | 0.101 |
| Pulmonary necrosis | 6 (3.5%) | 12（5.0%） | 0.524 | 0.469 |  | 1 (1.3%) | 2 (2.1%) | 0.178 | 0.673 |
| Pericardial effusion | 2（1.2%） | 4（1.7%） | 0.169 | 0.681 |  | 1 (1.3%) | 1 (1.0%) | 1.203 | 0.273 |
| Atelectasis | 13（7.6%） | 17（7.1%） | 0.047 | 0.829 |  | 6 (7.6%) | 12 (12.5%) | 1.164 | 0.281 |

Abbreviations: CRP, C‐reactive protein; LYM%, lymphocyte ratio; LDH, lactic dehydrogenase; M (IQR), median interquartile range; M (SD), median standard deviation; NEU%, neutrophil ratio; PCT, procalcitonin; PLT, platelet; WBC, white blood cell.
