# Supplementary material for: Development and validation of a clinical-radiomics nomogram for differentiating Mycoplasma pneumoniae pneumonia from bacterial pneumonia in children
Source: Front Pediatr. 2026 Jul 8;14:1764639. doi: 10.3389/fped.2026.1764639 (PMC13388284; doi:10.3389/fped.2026.1764639)
Supplement: Supplementary Figure S2 — (A) Chest CT of bacterial pneumonia (Streptococcus pneumoniae). Male, 62 months old, CRP 72 mg/L, WBC 12.8 × 109/L, LDH 380 U/L. Large areas of consolidation in the right upper lobe with visible air bronchograms. (B) Chest CT of mycoplasma pneumonia. Male, 56 months old, CRP 19 mg/L, WBC 10.9 × 109/L, LDH 291 U/L. Small patchy consolidations in both lower lobes and peribronchial ground-glass opacities (tree fog sign) in the right lower lobe. [file Table2.docx]

Table S2. Interobserver Agreement for CT Imaging Features

| CT Imaging Feature | Kappa (K) | 95%CI | *P* Value | Agreement Strength |
| --- | --- | --- | --- | --- |
| Left lung | 0.971 | 0.948-0.991 | <0.001 | Almost perfect |
| Right lung | 0.890 | 0.829-0.939 | <0.001 | Almost perfect |
| Bilateral lungs | 0.978 | 0.958-0.996 | <0.001 | Almost perfect |
| Bronchial wall thickening | 0.790 | 0.716-0.851 | <0.001 | Substantial |
| Interlobular septal thickening | 0.847 | 0.797-0.896 | <0.001 | Almost perfect |
| Tree-in-bud sign | 0.849 | 0.805-0.894 | <0.001 | Almost perfect |
| Tree fog sign | 0.825 | 0.767-0.875 | <0.001 | Almost perfect |
| Mediastinal or Hilar lymph node enlargement | 0.723 | 0.644-0.787 | <0.001 | Substantial |
| Consolidation extent | 0.941 | 0.884-0.940 | <0.001 | Almost perfect |
| Pleural effusion | 0.942 | 0.903-0.976 | <0.001 | Almost perfect |
| Pulmonary necrosis | 0.724 | 0.563-0.840 | <0.001 | Substantial |
| Pericardial effusion | 0.797 | 0.495-1.000 | <0.001 | Substantial |
| Atelectasis | 0.712 | 0.583-0.831 | <0.001 | Substantial |
