# Supplementary figures and images for: Development and validation of a clinical-radiomics nomogram for differentiating Mycoplasma pneumoniae pneumonia from bacterial pneumonia in children
Source: Front Pediatr. 2026 Jul 8;14:1764639. doi: 10.3389/fped.2026.1764639 (PMC13388284; doi:10.3389/fped.2026.1764639)

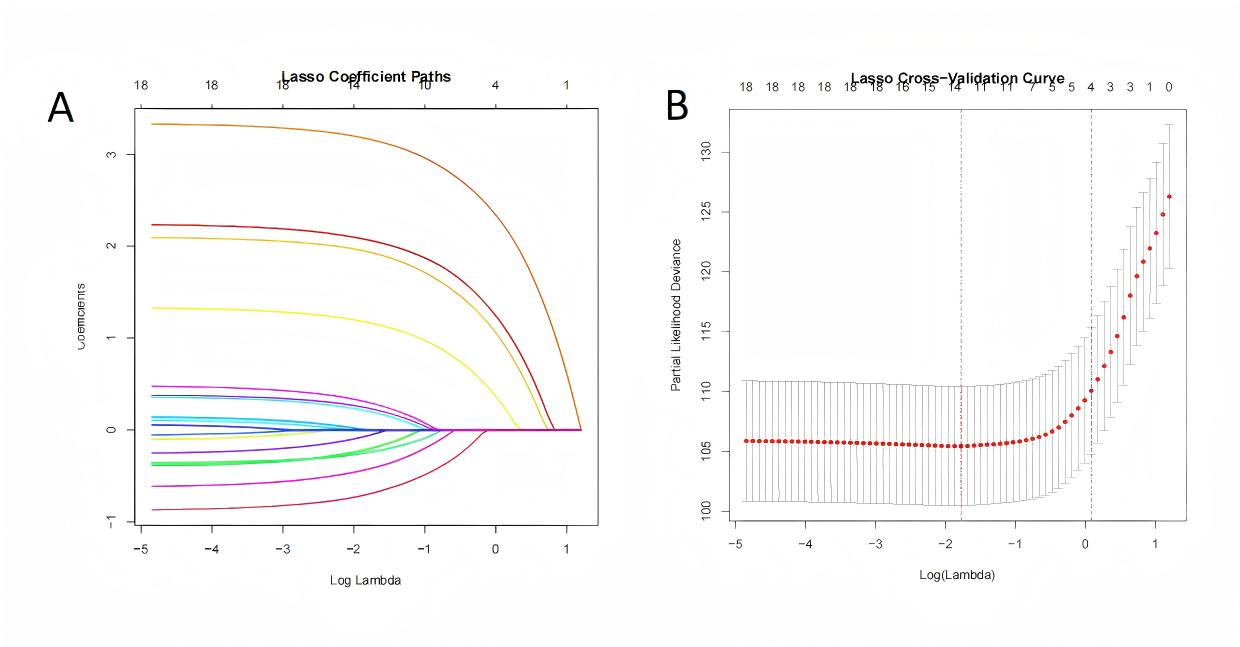

Supplement: Supplementary file 3 [file Image1.tif]

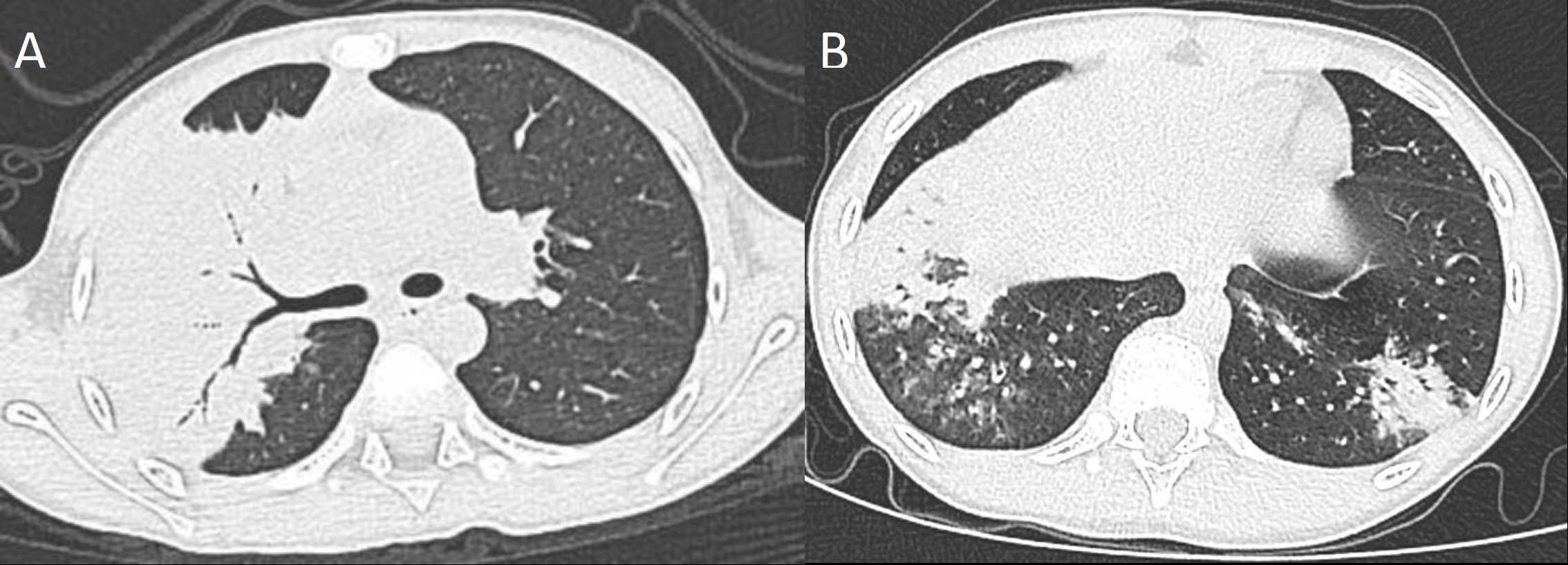

Supplement: Supplementary file 4 [file Image2.tif]
